# High-resolution microscopy and spectroscopy datasets meet *Data in Brief*

**DOI:** 10.1016/j.dib.2020.105596

**Published:** 2020-04-21

**Authors:** Stefano Coppola, Giuseppe Antonacci, Luca Lanzanò

**Affiliations:** a Division of Gene Regulation, The Netherlands Cancer Institute (NKI), Oncode Institute, Amsterdam, Plesmanlaan 121, 1066 CX Amsterdam, The Netherlands; b Photonics Research Group, INTEC, Ghent University-imec, Ghent 9052, Belgium; c Present address: Dipartimento di Fisica, Politecnico di Milano, Piazza Leonardo da Vinci 32, I-20133 Milano, Italy; d Dipartimento di Fisica e Astronomia "Ettore Majorana", Università degli Studi di Catania, Via S. Sofia, 64, 95123 Catania, Italy; e Nanoscopy and NIC@IIT, Istituto Italiano di Tecnologia, Via Melen 83, Genoa, Italy

We here introduce the Special Issue “*Optical microscopy and spectroscopy of single cells and molecules*” [1], which aimed to share high spatiotemporal resolution data collected with state-of-the-art microscopy and spectroscopy techniques. The data article format gives the opportunity to open access and reuse data whose acquisition and processing methods, including custom analysis scripts, are rigorously described and shared. The ultimate goal of the Special Issue is to contribute to the development and validation of new data interpretation models and analysis strategies for a better understanding of biological processes.

The Special Issue covers data acquired by several microscopy and spectroscopy techniques able to probe subcellular interactions, mechanical properties, and dynamics in living cells, organisms, and *in vitro* systems. The optical methods were based on fluorescence, inelastic Brillouin light scattering, or light diffraction.

Localizing single molecules at a spatial resolution below the diffraction limit is of utmost importance to understand their function at a subcellular scale. Among the several techniques developed to accomplish this task, stochastic super-resolution approaches utilize sequential activation and time-resolved localization of close-by photoswitchable fluorophores to create high-resolution images [2]. Points accumulation for imaging in nanoscale topography (PAINT) is a single-molecule localization method that achieves the stochastic single-molecule fluorescence by molecular adsorption/absorption and photobleaching/desorption. PAINT was initially limited to the environment-sensitive dyes and later extended to regular dyes (DNA-PAINT) [3]. In this Special Issue, *Delcanale et al.* provide single-molecule images of streptavidin-coated polystyrene particles using the DNA-PAINT imaging approach [4]. These data contain information about the spatial distribution of binding sites on the surface of individual particles and provide a reference for the quantitative characterization of functional nanomaterials using DNA-PAINT. Super-resolution optical fluctuation imaging (SOFI) is a post-processing method to obtain super-resolved images from image time series that is based on the temporal correlations of independently fluctuating fluorescent emitters, without the need to localize single fluorophores [5]. In this Special Issue, *Moeyaert et al.* describe a large dataset to assess the fitness of a broad panel (n=20) of fluorescent proteins for SOFI imaging [6]. The image time series were acquired under several imaging conditions and are a fundamental benchmark for further development of the SOFI algorithm and other stochastic super-resolution approaches.

In single particle tracking (SPT), the position of a fluorescently-labeled molecule or any other subcellular object is detected with nanometer precision as a function of time [7]. Trajectories are the most important type of data extracted from SPT experiments as their analysis can reveal complex mechanisms of motion. In this Special Issue, two data articles share image time series to investigate the 2D and 3D motion of fluorescently-labeled membrane proteins and mitochondria, respectively. *Mazloom-Farsibaf et al.* provide raw images of two different membrane proteins, i.e. the high-affinity IgE receptor FcɛRI, a transmembrane protein, and an outer-leaflet GPI-anchored protein [8]. The IgE receptor, labeled with Janelia Fluor 646, and the GPI-anchored protein, tagged using a GPI-GFP fusion protein and an ATTO 647N labeled anti-GFP nanobody, were imaged at high temporal resolution (490 Hz frame rate) in rat basophilic leukemia cells under control actin-stabilized (phalloidin) conditions. These raw images can be used for the development and testing of 2D SPT algorithms and to explore models of the actin cytoskeleton interaction with membrane proteins. *Mieskes et al.* provide the raw data for antero- and retrograde movement of individual mitochondria labeled with photo-activatable green fluorescent protein in the axon of sensory neurons in zebrafish embryos [9]. The trajectories were obtained using a 3D SPT method in which the laser excitation beam is rotated around the moving object, thanks to a feedback-based algorithm (Orbital Tracking) [10]. The data describe the movement of mitochondria with 10 ms temporal resolution and displacements of up to 100 µm and can be used by other researchers for developing detailed models of mitochondrial transport.

To investigate the dynamics and interactions of molecule ensembles in the intracellular environment, the spontaneous fluorescence intensity fluctuations are analyzed using fluorescence correlation spectroscopy (FCS) [11]. The raw data in FCS are represented by the fluorescence intensity as a function of time at high temporal resolution. In this Special Issue, two different types of FCS datasets were provided by two different labs. *Bathe-Peters et al.* share FCS data acquired on a commercial confocal microscope possessing a resonant scanner [12]. Data are acquired as sequential line-scans (temporal resolution < 100 µs) and analyzed to extract the diffusion coefficient of a dye in solutions of different viscosities and of a prototypical membrane receptor in live cells. The data are valuable for researchers interested in measuring the diffusion of nuclear, cytosolic or membrane-bound proteins using a commercial confocal microscope and can be used to test alternative algorithms for processing scanning FCS data. In another article of the Issue, *Karuka et al.* share FCS data taken at the nuclear envelope in live cells [13]. Measuring the mobility of proteins at the nuclear envelope is challenging with respect to other cellular compartments, as undulations of the envelope can produce slow fluctuations not related to the fast diffusion of the protein. The authors show how to process the raw data using the time-shifted mean-segmented function to isolate the fast fluctuations and measure the diffusion of a protein in the nuclear envelope. The data provide an opportunity to examine the influence of slow fluctuations on existing FCS data analysis methods. Raster image correlation spectroscopy (RICS) is a variation of FCS that exploits intensity fluctuations in images series acquired using a laser scanning confocal microscope [11]. In this Special Issue, *Lemmens et al.* provide two-color RICS data of the slowly diffusing Glycine membrane receptor (GlyRa3) recorded using a spectral detector [14]. To determine the diffusion coefficient and interaction strength, auto- and cross-correlations of eGFP and mCherry labeled GlyRa3 images were calculated. The data can be used as a reference for artifact-free two-color cross-correlation RICS that contains slowly diffusing interacting molecules.

At the nuclear level, several high-resolution microscopy methods have been developed to investigate the chromatin structure and compaction, gene expression, and mechanical properties of nucleic acids. In fluorescence lifetime imaging microscopy (FLIM) detection of Förster resonance energy transfer (FRET), the fluorescence lifetime of a fluorophore is measured at each pixel of an image to map the nanometer scale proximity with respect to another fluorescent molecule. A recent application of this technique is the study of chromatin compaction in a living cell via FRET between fluorescently labeled histones [15]. In this Issue, *Liang et al.* share FLIM data recording histone FRET in live cells co-expressing H2B-eGFP and H2B-mCherry [16]. The FLIM data, acquired in the frequency domain and processed by the phasor approach to lifetime analysis, provide insight into how to quantify FRET by phasor analysis in the presence of cellular autofluorescence. The data will benefit researchers interested in establishing the histone FRET assay in their specific cell system and will serve as a resource for learning the phasor approach to histone FRET analysis of chromatin compaction. Single-molecule fluorescence *in situ* hybridization (smFISH) has emerged as a powerful method to detect and count single nascent and mature RNAs at the single-cell level [17]. In this Special Issue, *Maekiniemi et al.* share raw and analyzed smFISH data of the cell cycle-controlled mRNA CLN2, in combination with immunofluorescence (IF) to quantify the protein expression of the cell cycle marker alpha-tubulin, in yeast cells [18]. These data, available as multi-color 3D z-stacks, can be used as a reference for setting up improved smFISH-IF protocols and validating new models to fit the mRNA distributions.

Single-molecule force spectroscopy by magnetic tweezers (MTs) is used to measure *in vitro* the mechanical properties of biomolecules [19]. The single-molecule (e.g. single-stranded or double-stranded DNA) is tethered on one end to a magnetic bead that can be displaced/rotated by a magnet and whose position, with respect to the tethering surface, is determined by its diffraction pattern. In this Special Issue, two data articles provide single-molecule MT datasets. *Vanderlinden et al.* share multiple high-resolution MT data of (i) the extension and torque of double-stranded DNA and RNA, (ii) the critical torque of buckling and the torsional stiffness of DNA and RNA as a function of applied force, and (iii) the hopping behavior at the DNA buckling point [20]. The provided datasets are a reference for experimentalists interested in DNA and RNA MT measurements and for testing and validating biomolecular simulations. *Ostrofet et al.* report data of the best achievable spatiotemporal resolution of MTs for DNA-tethered magnetic beads (0.3 nm steps along the z-axis), highlighting the influence of mechanical stability [21]. These data provide a useful benchmark for MTs setup development, adjustment, and optimization.

Lastly, Brillouin microscopy is an emerging spectral imaging tool holding great promise for mechanobiology and medical diagnostics [22]. The potential of this technique comes from its unique capability to assess the 3D biomechanical properties of cells and tissues in a non-contact and label-free manner, as opposite to standard invasive elastography techniques. Being purely optical, Brillouin microscopy can assess viscoelastic properties in intracellular compartments at high (optical) spatial resolution, heralding novel *in-vivo* applications for biomedical imaging [23]. In this Special Issue, we collected contributions from different research groups with the goal of sharing the raw data from which the spectra of the Brillouin light scattering are reconstructed. This will be of particular interest for all the existing and approaching researchers that seek to familiarize and increase awareness on the real data collected in Brillouin imaging experiments on diverse biological samples. *Sánchez-Iranzo et al.* present 3D measurements of Brillouin scattering spectra of the *in-vivo* zebrafish larvae eye [24]. This spectral dataset was obtained with 48-52 hours post-fertilization zebrafish embryos, encompassing the crystalline lens as well as several different retinal layers. This data provides a valuable resource as well as a starting point for researchers interested in the mechanobiology of vertebrate eye development. *Bailey et al.* presented data collected from gelatin hydrogels using a lab-built Brillouin microscope with a dual-stage VIPA spectrometer [25]. Gelatin hydrogels are of interest because they can be used as tissue-mimicking model systems for Brillouin microspectroscopy measurements. In this work, the authors varied the water concentration of the hydrogels and measured the corresponding evolution in the Brillouin-derived longitudinal elastic modulus measured, showing an increase in Brillouin frequency shift with increasing solute concentration. *Caponi et al.* collected data from a custom micro-spectrometer, which simultaneously acquires Raman and Brillouin spectra to analyze the chemical composition and the mechanical properties of single cells [26]. A dataset acquired on fixed NIH/3T3 murine fibroblast cells is reported. This will be valuable for researchers interested in the analysis of single cells by Raman and Brillouin spectroscopy to compare data acquired by different setups and to model the fitting functions correctly.

In conclusion, this Special Issue publishes detailed and valuable data acquired in real experiments with several microscopy and spectroscopy techniques that have seen increasing interest amongst different biomedical communities. It will be of particular usefulness to aid interested researchers in approaching those techniques and to raise awareness on the type of raw data being acquired in the respective labs.
